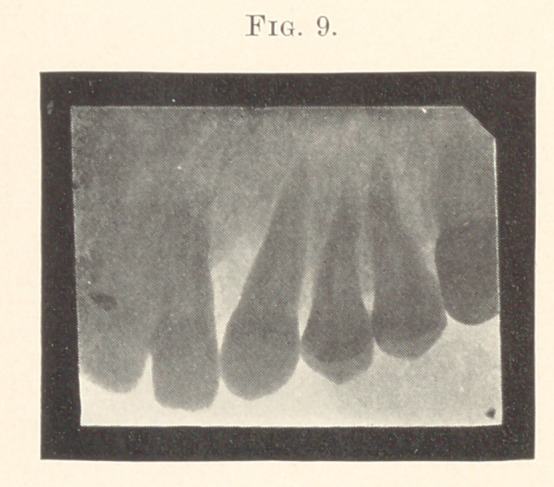# A Case of Orthodontia Diagnosed and Progress Watched by the X-Ray

**Published:** 1901-03

**Authors:** W. E. Decker

**Affiliations:** Boston, Mass.


### International Dental Journal.

Vol. XXII. March, 1901. No. 3.

### Original Communications.[1]

### A CASE OF ORTHODONTIA DIAGNOSED AND PROGRESS WATCHED BY THE X-RAY.[2]

BY W. E. DECKER, D.D.S., BOSTON, MASS.

Mr. President and Gentlemen,—I appreciate very much the honor of appearing before the American Academy of Dental Science, and I trust I may be able to interest you for a few moments.

Many of the details of this case of regulating are not of particular interest. The all-important point is set forth in the subject of the paper,—"A Case of Orthodontia diagnosed and Progress watched by the X-Ray."

As Dr. Angle said at Providence, a short time ago, "Many of our cases fail, or are more difficult, because of a lack of a complete and correct diagnosis." We often study them in a short-sighted way, and endeavor to correct only the most conspicuous wrong, and, of course, fail to establish a correct occlusion and harmony between the arches and in the facial lines.

"The truth, the whole truth, and nothing but the truth" is

[1] The editor and publishers are not responsible for the views of authors of papers published in this department, nor for any claim to novelty, or otherwise, that may be made by them. No papers will be received for this department that have appeared in any other journal published in the country.

[2] Read before the American Academy of Dental Science, December 5, 1900.

absolutely necessary for an intelligent and complete treatment. Previous to the introduction of the X-ray in dentistry the "whole truth" was sometimes impossible. But now with the skiagraph (and I hope some day with our own eyes) we are able to come very near it.

The patient was a girl fourteen years old. (Fig. 1.) The irregularity of the teeth was so great as to cause a marked deformity in the face. At first thought it would be pronounced a case of orthognothism, which, however, is only seeming. The slight protrusion of the chin is aggravated by a lack of development in the superior maxilla. A straight line touching the frontal and mental prominences shows the nose and upper lip to be much too far back. Upon looking at the bite the cause is obvious. (Fig. 2.) Two teeth are wanting in the superior arch. There is no sign of the left cuspid, the lateral standing in contact with the first bicuspid. On the right side the cuspid stands out on the labial gum some distance above its proper position, and the lateral and bicuspid are nearly in contact. Note the absence of any prominence on the gum to suggest an impacted cuspid. As you see by the models, our landmarks, the first molars occlude almost correctly. On the left side the superior first molar occludes about one-third of a tooth distally. On the right side the occlusion of the first molars, mesodistally, is almost correct. Please note the thinness of the plate over the anterior teeth.

The cause of the deformity seems to be an inherited tendency. It is a prominent characteristic of the family through several generations. Out of thirty-five persons in the last four generations, twenty-three are afflicted and only twelve have escaped, and even in these the suggestion is evident. Fig. 3 is a model of another member of the family. It is not only an unsightly deformity, but also a severe detriment to health. The incisors of the upper arch pass far below those of the lower arch and stand three-eighths of an inch inside the proper line of occlusion. Only five teeth meet for the mastication of food. Probably this would have been the fate of our patient had not orthodontia come to her rescue.

(Fig. 4.) When the patient first presented for consideration, it was thought by the family and myself that the superior left cuspid had been extracted. Other dentists, also, were of the same opinion, arguing that the arch had fallen, the lateral hurriedly

Fig. 1.

Fig. 2.

Fig. 3.

Fig. 4.

tipping into the cavity left by the extraction. There was no prominence on the roof of the mouth to show the presence of the tooth.

To improve the condition of the case without another tooth, either natural or false, was quite impossible. Therefore it was highly necessary to have positive proof of the absence or presence of the cuspid. The X-Ray quickly clears up these hidden mysteries. (Fig. 5.) And, as we see by the skiagraph, the cuspid lay deeply hidden in the bone. Its exact position is thus clearly shown. It stood at an angle of about forty-five degrees. The cusp was behind the lateral, about half-way up on the root. The apex of the root was about an eighth of an inch directly above the second bicuspid. The skiagraph told us that it was palatal to the lateral, as the outline of the cusp is slightly clearer than that of the lateral root at the point of lapping, showing that the cuspid was nearer the film.

We took several pictures,—one with the tube farther in front of the patient, another with the tube farther back, while this one was taken directly in front of the angle of the jaw. By comparing the negatives, we further learned that the cuspid was not rotated in its pocket but lay with its labial side out. Now, you see, we had that part of the case completely and correctly diagnosed, and what would probably have always remained a mystery is thus by the aid of the X-ray fully comprehended.

As I have said before, our first molars were in about correct occlusion, so the case did not call for the retraction of the chin, although a slight movement in this direction might have been beneficial. The course of treatment was now to expand the arch somewhat, slightly rotate the left first bicuspid, protrude the left lateral and central the width of a cuspid and the right central and lateral half that width, to elongate and press into line the right cuspid, and to erupt and bring into line the embedded left cuspid, and this is how I proceeded:

I banded the first molars with adjustable clamp bands and slipped into the tubes the expansion arch under tension. Then I ligated all of the teeth to the arch in a certain rotation. First the arch was pressed up and the right cuspid ligated to elongate. Then the four bicuspids were wired to expand and the incisors to protrude. While the tension of the wire arch was elongating the cuspid and expanding the arch, the protrusion was accomplished by constantly tightening the nuts on each side, the left considerably more than the right. I did not attempt to make any speed with the case. In fact, the alveolar plate over the anterior teeth was so thin that I was compelled to go slowly in order to give nature time to build new bone. For a short time I had a band on the first bicuspid, with a small ring on the labial surface. Between this ring and the lateral I placed a small jack-screw, which rotated the bicuspid and at the same time helped to hurry forward the left incisors.

In five weeks I had the desired space, as this model shows. (Fig. 6.) Please again note the exceedingly thin anterior alveolar plate. For this radical change I was severely criticised, some contending that the teeth protruded in an unsightly manner. And so they did, but nature and our fulcrum retainer would not leave them so. Our active labors were finished for a time. What we had done was to help or encourage nature, and the retainer was so constructed that a constant improvement would gradually go on. It was practically a lever of the first class, the lips being the power, the wire to which the teeth were attached the fulcrum, and the process the weight. The manner of the attachment was the secret of the success. The right first molar was banded with a tube on the buccal surface, as was also the left first bicuspid that had been rotated. Upon the four centrals, high up at the gum-line, were cemented gold bands, on the labial surfaces of which were small tubes that had previously been split open. A wire arch was then placed in all these tubes. As it entered those on the side teeth it fell into the open ones on the anterior teeth; then they were pinched shut around the wire, leaving the teeth to swing freely on this as the fulcrum. I wish to here point out the great advantage of this over any rigid attachment. If all the bands had been soldered together, or all soldered to the wire arch, the teeth would have had to remain as they were, which would have left the case in a bad predicament, as the protrusion was so conspicuous. As it was, however, the teeth could move freely on the wire, as a fulcrum, and the lip was the power that in a few months brought about a wonderful and gratifying change. Its pressure tipped the roots forward, the process gradually filling out at the base of the nose to a remarkable extent, giving a vast improvement to the profile. The other teeth were attached to the arch by wire ligatures for a time, and a false cuspid fastened to the arch in the new space. Broken bits of the retainer can be seen on the model.

Fig. 5.

Fig. 6.

Fig. 7.

Fig. 8*a*.

Fig. 8*b*.

Fig. 9.

The skiagraph (Fig. 7) taken at this time shows the cuspid unlocked from behind the lateral root and standing directly above interposed false tooth.

Here we rested for one year. During the interval I saw the patient but twice. The cuspid had not made its appearance, and we concluded to forcibly erupt it. A skiagraph (Fig. 8*a*) showed that it had started but very little, and might have erupted in a few years. However, it would be impracticable to hold the space that long. During the year I lost some of the space,—not as much as appears in the negative, however, as this was not taken directly in front, and the shadow of the bicuspid extends into the opening.

Knowing exactly where to find the tooth, after an injection of eucaine I lanced the gum and chipped away a thin layer of process, finding a beautiful, well-developed tooth. I then drilled a small pit into the centre of it at the gingival border, threaded the cavity with a How tap, and screwed in a small post. (Fig. 8*b*.) The expansion arch was replaced and a wire ligature carried from the post down about the arch. By constantly twisting the wire, which put the arch under tension, the elongation went on rapidly. In the negative the post, arch, and twisted wire can be plainly seen. I also had a small hook thrown behind the lateral, and by screwing a nut on the arch straightened that tooth somewhat. In twenty-one days the tooth was brought down to its proper position, except that it was a little palatal to the correct occlusion. A longer-threaded post in the tooth, with a large flat nut that screwed against the outside of the arch, soon brought it labially as far as desired. (Fig. 9.)

As an acquaintance of mine often says, he would rather regulate fourteen times than retain a case. So I began to think before I found a retainer that would successfully hold the tooth in the arch. Of course, it was very loose and seemed to be hung on elastics, and would jump back the moment it was released. I made several retainers before I succeeded. The final one (Fig. 10) was a gold band around the cuspid, wide on the palatal side but narrow and under the gum on the labial surface, soldered to an adjustable band on the bicuspid, both being cemented on. To hold the expanded arch I constructed one of Dr. Baker's plates, with the small wires touching each tooth. These, resting against the incisors well up at the gingival border, acted as fulcrums, and the lips are constantly improving the position of the teeth.

The difference in the bite before and after the treatment is conspicuous. (Compare Figs. 2 and 11.) The one shows quite a normal occlusion,—at least a harmony between the arches. The impression for this model was taken recently, only four months after the completion of the work. The patient is still wearing the retainers. In a year or two from now I expect to find the position of the centrals still better and the depression at the base of the nose entirely gone.

The inside of the arch, also, shows a marked change. (Compare Figs. 4 and 12.) It is larger in every way. All of the teeth are in their places, the general contour is graceful, and the usefulness of the arch is established. In all probability the nasal fossæ are enlarged, and the patient is less liable to have adenoids, catarrh, and other nasal difficulties.

The profile is also improved. (Compare Figs. 1 and 13.) The chin does not seem to be so prominent. The face is more balanced, and will continue to improve instead of becoming worse as she grows older, as it surely would have done without any interference.

I am greatly indebted to Dr. D. M. Clapp for the X-ray work. The films are all very good, showing much more detail than can be reproduced in a cut.

## Figures and Tables

**Fig. 1. f1:**
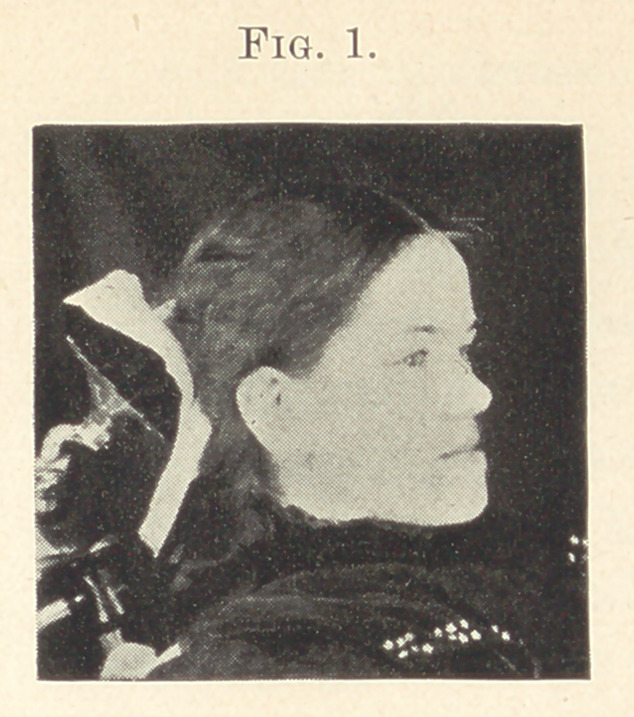


**Fig. 2. f2:**
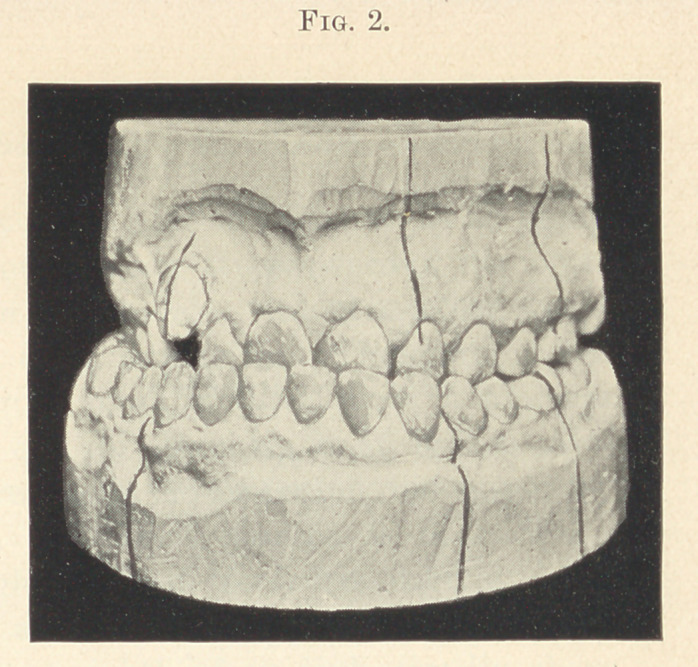


**Fig. 3. f3:**
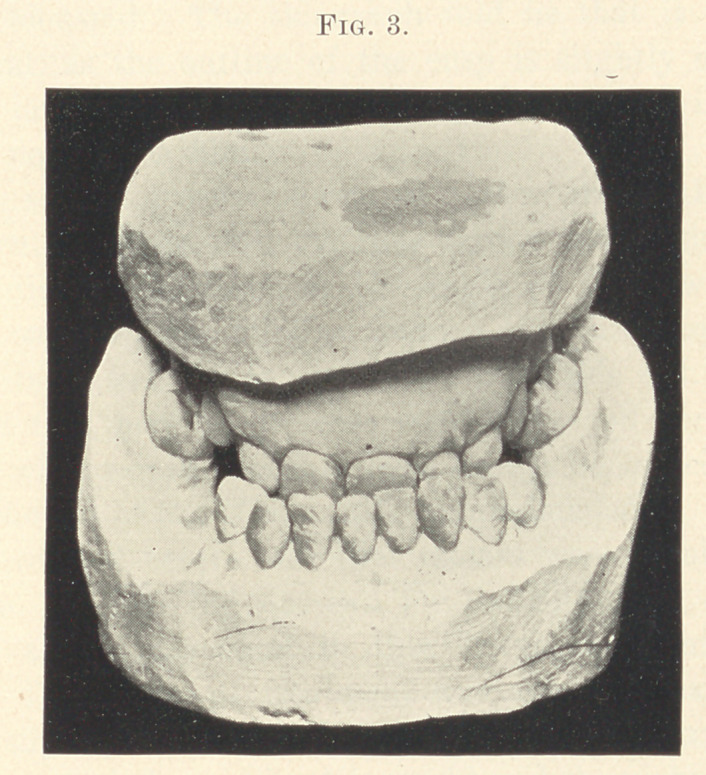


**Fig. 4. f4:**
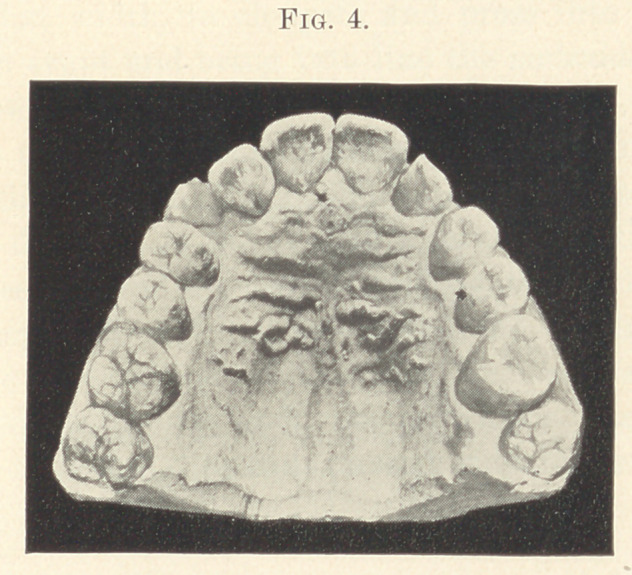


**Fig. 5. f5:**
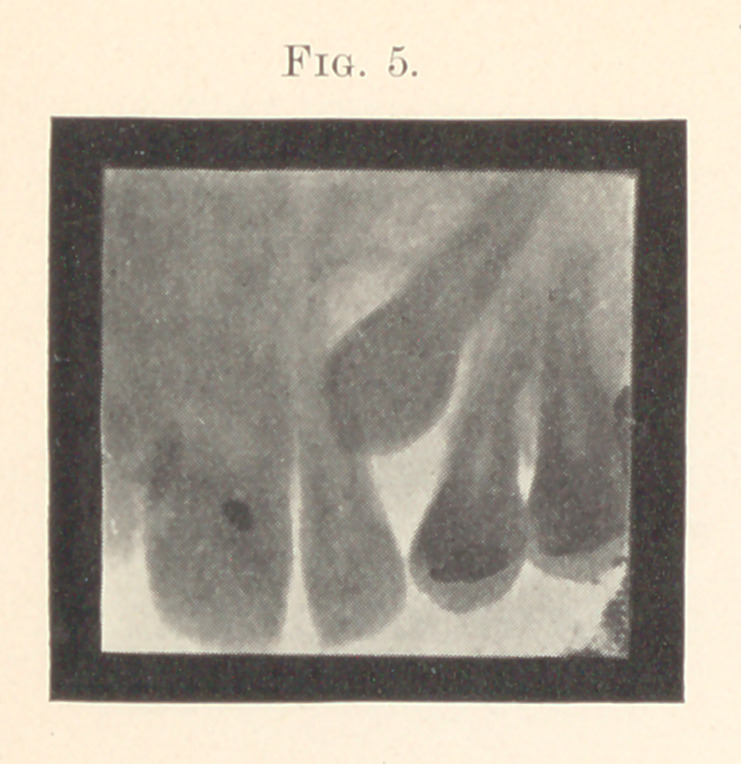


**Fig. 6. f6:**
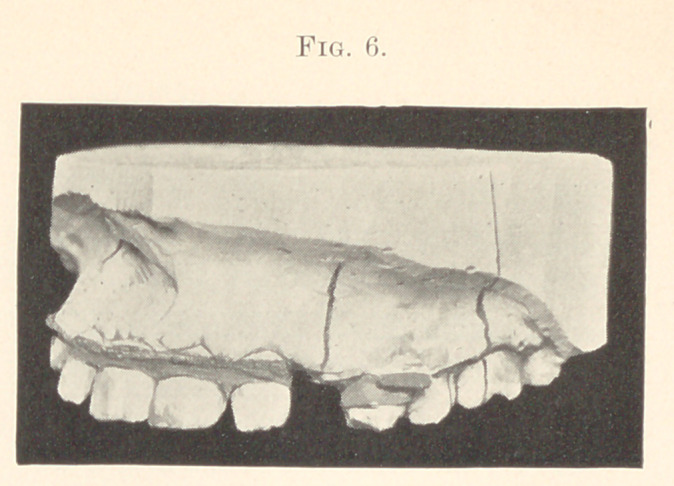


**Fig. 7. f7:**
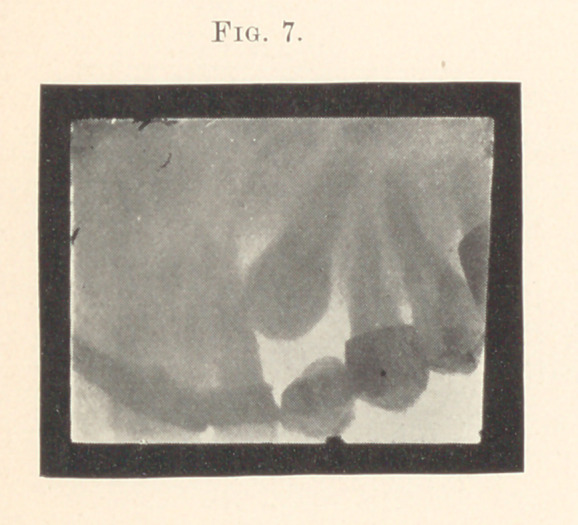


**Fig. 8a. f8:**
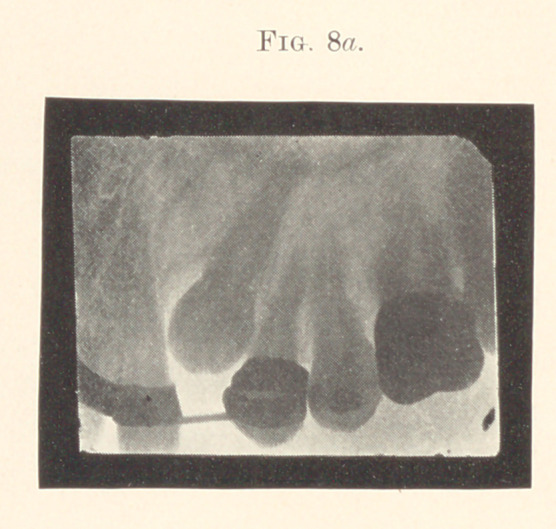


**Fig. 8b. f9:**
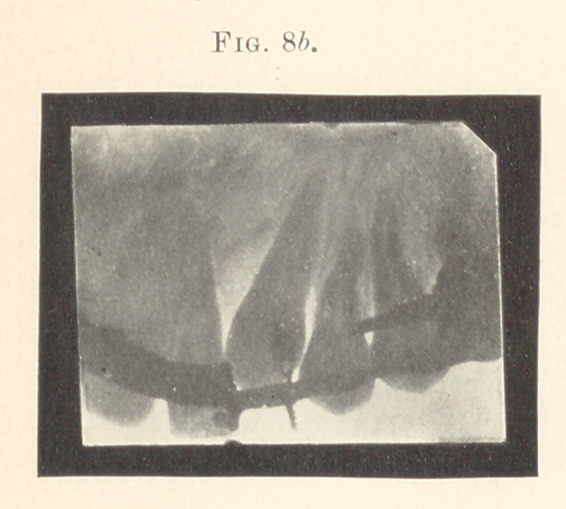


**Fig. 9. f10:**